# Frailty as a growing challenge for anesthesiologists – results of a Dutch national survey

**DOI:** 10.1186/s12871-021-01528-x

**Published:** 2021-12-06

**Authors:** A. Bouwhuis, C. E. van den Brom, S. A. Loer, C. S. E. Bulte

**Affiliations:** 1 Departments of Anesthesiology Amsterdam UMC, VU University, Amsterdam Cardiovascular Sciences, De Boelelaan 1117, 1081 HV Amsterdam, The Netherlands; 2 Departments of Intensive Care, Amsterdam UMC, VU University, Amsterdam Cardiovascular Sciences, De Boelelaan 1117, 1081 HV Amsterdam, The Netherlands; 3 Department of Intensive Care, Amsterdam UMC, University of Amsterdam, Amsterdam, the Netherlands

**Keywords:** Aging, Anesthesiologist, Elderly, Frailty, Management, Netherlands, Organization, Perioperative, Survey

## Abstract

**Background:**

Frailty is a multidimensional condition characterized by loss of functional reserve, which results in increased vulnerability to adverse outcomes following surgery. Anesthesiologists can reduce adverse outcomes when risk factors are recognized early and dedicated care pathways are operational. As the frail elderly population is growing, we investigated the perspective on the aging population, familiarity with the frailty syndrome and current organization of perioperative care for elderly patients among Dutch anesthesiologists.

**Methods:**

A fifteen-item survey was distributed among anesthesiologists and residents during the annual meeting of the Dutch Society of Anesthesiology. The first section included questions on self-reported competence on identification of frailty, acquaintance with local protocols and attitude towards the increasing amounts of elderly patients presenting for surgery. The second part included questions on demographic features of the participant such as job position, experience and type of hospital. Answers are presented as percentages, using the total number of replies for the question per group as a denominator.

**Results:**

A sample of 132 surveys was obtained. The increasing number of elderly patients was primarily perceived as challenging by 76% of respondents. Ninety-nine percent agreed that frailty should influence anesthetic management, while 85% of respondents claimed to feel competent to recognize frailty. Thirty-four percent of respondents reported the use of a dedicated pathway in the preoperative approach of frail elderly patients. However, only 30% of respondents reported to know where to find the frailty screening in the patient file and appointed that frailty is not consistently documented. Interestingly, only 43% of respondents reported adequate collaboration with geriatricians. This could include for example a standardized preoperative multidisciplinary approach or dedicated pathway for the elderly patient.

**Conclusions:**

This survey demonstrated that the increasing number of frail elderly patients is perceived as important and relevant for anesthetic management. Opportunities lie in improving the organization and effectuation of perioperative care by more consistent involvement of anesthesiologists.

**Supplementary Information:**

The online version contains supplementary material available at 10.1186/s12871-021-01528-x.

## Background

Frailty is a multidimensional condition characterized by loss of functional reserve, loss of homeostatic mechanisms and increased vulnerability to adverse outcomes following stressors such as surgery [1]. The number of frail elderly is growing as the population is aging. Worldwide, the population over 60 years of age will nearly double from 12 to 22% between 2015 and 2050 [2]. As a consequence, the number of frail patients presenting for specialized medical care and hospital admission will even further increase. The prevalence of frail individuals in society is estimated at 4,1-8,5% while frailty is present in 10-37% of patients in a general surgery population [3, 4]. The aging population is paralleled by increased health care costs and in the Netherlands, it is expected that in 2030 58% of the national health care budget is spent on patients over 65 years of age [5, 6].

Aging decreases physiological reserve and increases incidence of disease, resulting in numerous comorbidities and polypharmacy [7]. While it is generally accepted that frailty prevalence increases at higher age, it is not an inevitable consequence of aging. In general, surgery in elderly patients has higher complication rates than in younger subjects. Adverse outcomes are mainly related to comorbidity, polypharmacy, impaired functional status, cognitive and sensorial disabilities rather than to chronological age. The preexisting presence of a geriatric syndrome is of importance since interventions might be available for preoperative optimization. Specifically in case of frailty, it is known to be a better predictor of health than separate comorbidities [1]. Frailty increases the odds of perioperative adverse outcome, such as increased morbidity, mortality, readmission rates, length of hospital stay and decreased self-reliance and quality of life [3, 8]. Adverse outcomes can be reduced when risk factors are recognized early, especially when dedicated care pathways are in place tailored at avoiding major adverse events during critical time points before and after interventions [9]. Of major importance is that these pathways should include frequent risk assessments by dedicated personnel, allowing patients to be transferred to a different pathway if necessary. Early recognition of frailty is of key importance in which anesthesiologists could play a crucial role.

Frailty is a modifiable risk factor for the recovery of patients after surgery. A variety of studies confirm improved outcomes after initiation of multimodal interventions including prehabilitation programs in frail subjects [10]. However, optimization can only occur when patients at risk are identified early and treated accordingly [11]. Traditional risk assessment is predominantly organ-system based and applying a similar method in elderly individuals will insufficiently identify those at high risk of frailty. Patients with frailty require a comprehensive perioperative approach. The focus should shift from handling disease to preserving performance and quality of life should be centralized [12].

The anesthesiologist beholds a key role in early identification of risk factors for frailty and preoperative optimization. It is, however, unknown whether anesthesiologists are familiar with the frailty syndrome and how they incorporate this knowledge into daily practice. We therefore investigated the perspective on the aging population, familiarity with the frailty syndrome and the current organization of perioperative care for elderly patients by conducting a survey among anesthesiologists and residents in the Netherlands.

## Methods

### Study population

The survey was distributed on the first day of the annual meeting of the Dutch Society of Anesthesiology in May 2019. All visitors were informed about the research activities during the first plenary session. Participants were either volunteering spontaneously or asked to participate by members of the research team. Only participants in possession of an active medical license as a physician or physician assistant and at the time of the survey working in the Netherlands were included. This study did not involve patients and did not subject participants to any conditions other than a short and one-time survey without questions of sensitive nature. Therefore, this survey is not covered by the Medical Research Involving Human Subjects Act (WMO) as described in Article 1b [13]. As a consequence, formal approval of the Institutional Ethics Board was not required by Dutch law. Written informed consent as described in WMO Article 1v was therefore not obligated [13]. All participants were however fully informed about the nature and purpose of the survey and provided verbal informed consent prior to filling out the survey. After data collection responses were anonymized.

### Questionnaire

A fifteen-item paper survey was designed by the authors, because no validated instrument fulfilled the objective. The full questionnaire can be found in the supplement [see supplement A]. The first section included closed-ended questions on self-reported competence on identification of frailty, acquaintance with local protocols and attitude towards the increasing amounts of elderly patients presenting for surgery. The second part included questions on demographic features of the participant such as job position, experience and type of hospital.

### Data extraction and statistical analysis

As a result of the explorative nature of the survey no power calculation was conducted beforehand. The collected data was entered and analyzed by the first author in Microsoft Excel (Version 16 for Mac, Microsoft Corp, Redmond, Washing, USA), after which the responses were anonymized. Following data extraction, answers were coded as true or false (or unknown). If multiple answers were given the total number of replies to each question was used as the denominator. Incomplete forms were not excluded. Opinion-based and demographic variables were presented as categorical variables. Graphs were made using Graphpad Prism (Version 8, Graphpad Software, San Diego, California, USA). The full data extract is added as additional material [see supplement B].

## Results

### Respondents

**Fig. 1:**
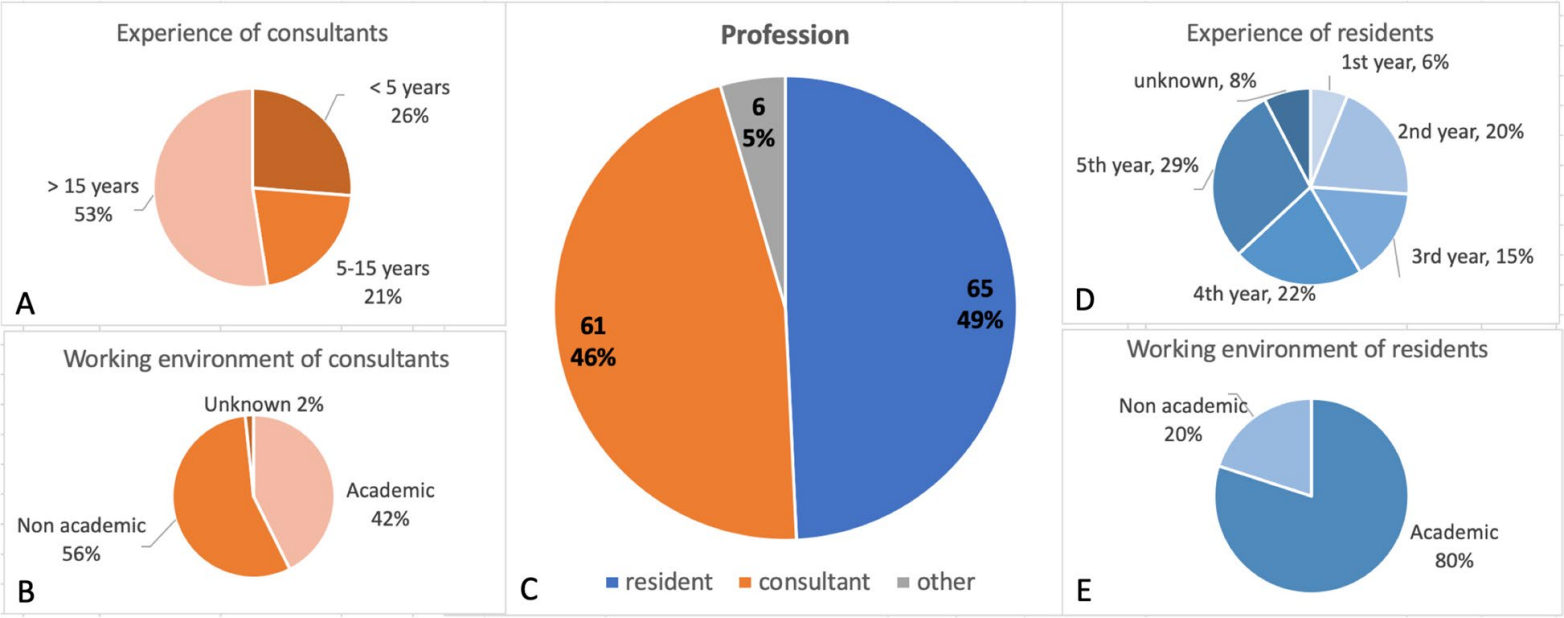
Characteristics of survey respondents. Experience level of consultants (orange, **A**), work environment of consultants (orange, **B**), total population divided by consultants in orange and residents in blue (**C**), experience of residents (blue, **D**) and work environment of residents (blue, **E**)

The meeting was attended by 1394 members of the Dutch Society for Anesthesiology. In total, 132 surveys were completed, resulting in an overall response rate of 9,5%. The respondents included 65 residents (49%), 61 anesthesiologists (46%) and 6 ‘others’ (5%) consisting of 2 physician assistants and 4 respondents with unknown profession (Fig. 1).

Of the consultants, 26% reported less than 5 years of experience, 21% between 5 and 15 years, and 53% over 15 years of experience (Fig. 1A). Forty-two percent of consultants worked in an academic setting versus 56% in a non-academic setting (Fig. 1B). Of the residents, 6% of respondents included first year residents, 20% second year, 15% third year, 22% fourth year and 29% fifth year (Fig. 1D). Eighty percent of the residents worked in an academic setting versus 20% in a non-academic setting (Fig. 1E). Respondents were working in 26 different hospitals in the Netherlands.

### The aging population and the anesthesiologist

**Fig. 2:**
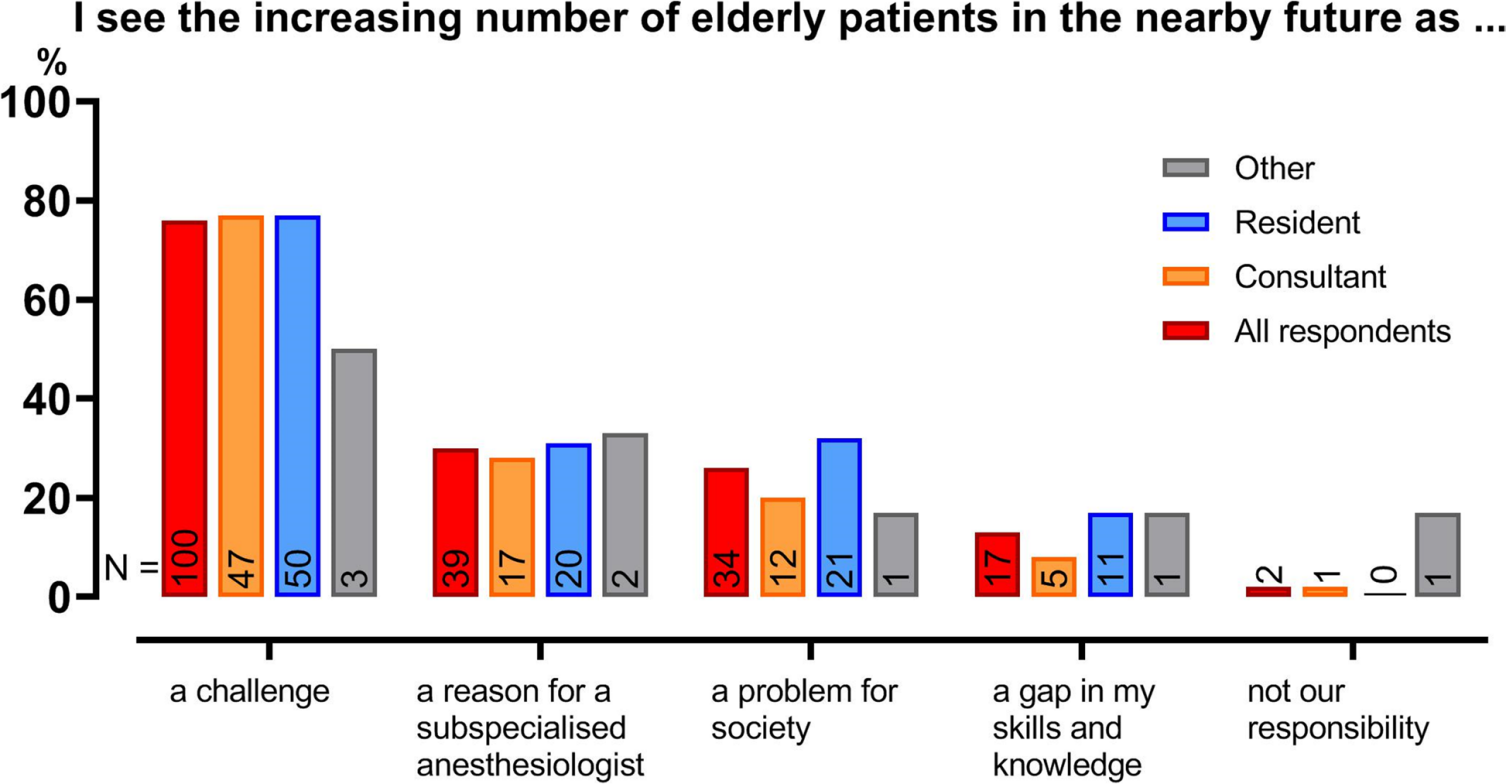
Opinions of respondents on increasing number of elderly patients. In the current question multiple statements were allowed. The red bar indicates the total respondents agreeing with the statement, which were subdivided in consultants (orange bars), residents (blue bars) and others (gray bars). The number of respondents is presented within the bars, and the percentage of respondents per group is presented on the y-axis

The increasing number of elderly patients was primarily perceived as a challenge by 76% of the respondents (Fig. 2). Further, 30% of the respondents perceived the increase in elderly patients as a reason for an anesthesiologist with elderly patients as subspecialty, whereas 26% of the respondents stated the increase as a problem for the society as a whole. Additionally, 13% mentioned the growing number of elderly patients to indicate a gap in their skills and knowledge regarding their perioperative management. Residents endorsed this statement twice as frequent compared to consultants (17% of residents versus 8% of consultants). For this question, multiple statements could be endorsed per respondent.

### Frailty and the anesthesiologist

The opinions of anesthesiologists regarding frailty are summarized in Table 1. Ninety-eight percent of respondents agreed that the anesthesiologist should be aware of the presence of frailty and 99% agreed it influences perioperative anesthetic management. Furthermore, 85% claimed to feel competent to recognize frailty in elderly. However, solely 39% of respondents report the presence of frailty in the patient file during the preassessment visit (23% of residents versus 52% of consultants). Moreover, only 30% seemed aware that all patients over 70 years of age currently receive a frailty screening on hospital admission and admitted to know where to find the documented frailty screening in their hospital (14% of residents and 41% of consultants).

**Table 1:** Opinions of anesthesiologists regarding frailty

Statements (***N*** = 132)	Agree (%)
The anesthesiologist should be aware of the presence of frailty	98%
The presence of frailty should influence anesthetic management	99%
I feel competent to recognize frailty	85%
During preassessment, I report the presence of frailty in the patient file	39%
All patients > 70 years of age are screened for frailty when entering the hospital	30%
In my hospital, I know where to find the frailty screening in the patient file	30%

Numbers show the percentage of respondents that agreed with the statement

### Organization of multidisciplinary geriatric care

Regarding multidisciplinary care, 85% of respondents claimed the availability of geriatric expertise at their hospital. The majority of respondents (54%) feel that the geriatrician is considered to be in the lead. The second most common scenario, as marked by 29% of the respondents, is that the surgical specialist consults the geriatric department. Sixteen percent of respondents reported that the geriatrician is consulted by the anesthesiologist themselves. Responses concerning the manner of consultation were independent of respondents’ position or work environment.

**Fig. 3:**
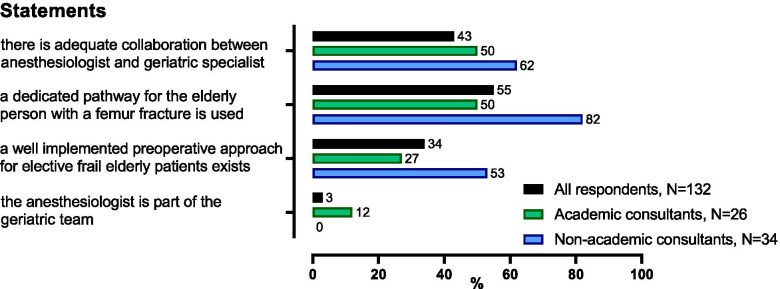
Influence of work environment on multidisciplinary geriatric care. Bars show the percentage of respondents that agree with the statement. The black bar represents all respondents; followed by consultants from academic centers (green bars) and consultants from non-academic centers (blue bars) who agree with the statement

Considering collaboration with geriatric specialists and the implementation of dedicated pathways, all received inferior satisfactory rates by the respondents from academic centers compared to colleagues from non-academic hospitals (Fig. 3). Due to the large portion of residents in academic work environments, comparisons were made between consultants only. Multidisciplinary dedicated care pathways for elderly patients originated from hip fracture repair procedures with high morbidity and mortality, which remain the most common performed procedure in often multimorbid complicated patients. Interestingly, only 50% of academic consultants reported the use of a dedicated pathway for elderly with a femur fracture (compared to 82% of nonacademic consultants). Similarly, only 27% of academic consultants reported a well implemented preoperative approach for elective frail elderly (compared to 53% of nonacademic consultants). The collaboration with geriatricians is reported as adequate by only 43% of all respondents. The presence of an anesthesiologist in the geriatric team is rare in both settings (3% in total; of which 12% of academic and 0% in non-academic centers).

## Discussion

The results from this survey demonstrate that anesthesiologists and residents in the Netherlands are familiar with the frailty syndrome, and agree that frailty influences perioperative anesthetic management. The increasing number of frail elderly patients is perceived as important and relevant for anesthesiologists, however, also as a challenge. Unfortunately, there is low familiarity with the current Dutch frailty screenings among the respondents, and frailty is infrequently documented during preassessment visits. The presence of dedicated geriatric care pathways, participation of anesthesiologists in multidisciplinary geriatric teams and collaboration with geriatric specialists is limited and generally not perceived as well implemented, especially in academic hospitals. Altogether, the challenge lies in improving the organization and effectuation of perioperative care for elderly patients.

The present study shows that the increasing number of frail elderly patients is important and relevant for anesthesiologists, however, that the participation of anesthesiologists in perioperative care for these patients in the Netherlands is currently limited. This is in line with a previous study concluding that involvement of anesthesiologists varies dependent on how important and involved people feel towards multidisciplinary anesthetic geriatric perioperative care for frail elderly [14]. Also, a recent edition of Anesthesia & Analgesia on frailty called anesthesiologists for action to better appreciate how perioperative outcomes are impacted by frailty by increasing awareness, education, geriatric collaboration, dedicated care pathways, and incorporation of the perioperative setting into frailty research [15]. Similarly, the guideline from the AAGBI states there is an expanding role for anesthesiologists into multidisciplinary perioperative care for the elderly [16]. This is confirmed by the present study by the unanimous opinion of the respondents stating that anesthesiologists should not only be aware of the presence of frailty, but also feel that the presence of frailty influences anesthetic management. The more active role for anesthesiologists appears rational as perioperative derailment of physiology and development of delirium is greatly influenced by various aspects of anesthetic practice. Elements that impair postoperative recovery include altered consciousness, immobility, pain, fluid disturbances, hypothermia, altered appetite and the substantial number of medications [17]. Several optimization strategies could be applied by the anesthesiologist. The preoperative evaluation should preferably occur at least 1 to 3 weeks prior to surgery via face-to-face visits in the presence of a relative or care-giver to gather important information about functional status. Timely evaluation creates the possibility for medication review including substance abuse, geriatric consultation, or the initiation of prehabilitation regimens aiming at correction of anemia, nutritional status, exercise capacity, cognitive or social support. Furthermore, logistics regarding the procedure (e.g., procedure in day care setting or hospitalization, postoperative ICU admission) and choice for anesthetic technique and monitoring should be tailored to the identified most profound vulnerabilities. Thorough preoperative evaluation clarifies benefits and risks, which should not be limited to the procedure and anesthesia alone, but also incorporate the expected influence on postoperative functional status. Preoperative knowledge of the presence of frailty will enable patients, their families, anesthesiologists, intensivists and surgeons to make informed decisions regarding treatments and surgical options. Taken together, these findings suggest a role for anesthesiologists in perioperative management of frail elderly patients.

Another finding of the present survey is that frailty is perceived as a responsibility of all clinicians, including anesthesiologists, but more education is desirable to gain confidence in frailty assessment. Deficiencies in training and resources as a barrier to identification of frailty in hospitalized patients were already reported in the United Kingdom [18]. A survey amongst health care professionals in Canada also stated that the lack of knowledge about frailty was a prominent barrier to the use of frailty assessments in practice, despite clinicians’ understanding that frailty affects their patients’ outcomes [19]. We can therefore conclude that more education will improve timely recognition of the frailty syndrome in elderly patients.

Besides education on the assessment of the frailty syndrome, collaboration with geriatric specialists is of importance. In the current study unsatisfactory collaboration with geriatric specialists was identified as well as lack of knowledge on the presence of dedicated geriatric care pathways. Dutch guidelines recommend implementation of dedicated care pathways for frail elderly undergoing surgery in which multidisciplinary evaluation, monitoring and treatment by professionals with both surgical and geriatric expertise are essential to achieve favorable recovery [20]. The guideline suggests that frailty screenings by anesthesiologists would be sensible, followed by more elaborate assessment performed by geriatricians. Geriatric evaluation should occur early in the perioperative track [21]. and should include physical, cognitive and social domains. Besides medical history, functional status, cognitive and sensorial impairments, mood, living situation, social support and financial constraints influence the patients’ health status and resilience after surgery. It should also be reviewed what defines the patients’ quality of life in the current state and the near future. Currently, Dutch guidelines do not specify who should provide the perioperative geriatric evaluation; in the Netherlands geriatric consultation is most frequently performed by geriatric physician assistants or geriatric specialists. Global European guidelines on perioperative frail elderly patients are lacking. However, the guideline focusing on postoperative delirium, with frailty as a substantial risk factor, also encourages education and implementation of multidisciplinary team-based approaches [22]. Co-management by geriatric specialists has been proven to improve patient outcome in patients with hip fractures as well as other procedures [23]. Taken together, more consistent participation of anesthesiologists in multidisciplinary geriatric teams and collaboration with geriatric specialists are opportunities for optimization of perioperative care of frail elderly.

This study has several limitations. First, there was a low response rate and although respondents were from hospitals throughout the Netherlands and included academic as well as non-academic centers significant response bias arises. Respondents that already have interest in geriatric care may have been more likely to respond to the survey. Secondly, the comparison between subgroups could have been affected by the small sample size and the relatively large number of residents. Residents naturally have fewer years of perioperative experience, therefore these results need to be interpreted with caution.

## Conclusions

This study provides insight into the perspective on the aging population, familiarity with the frailty syndrome and current organization of perioperative care for elderly patients by anesthesiologists in the Netherlands. The increasing number of elderly patients is perceived as a challenge. Furthermore, the respondents unanimously agreed that the anesthesiologist should be aware of the presence of frailty and that this influences anesthetic management. Despite the widely-recognized importance of integrating frailty into perioperative care, the challenge lies in improving the organization and effectuation of perioperative care for frail elderly patients. The present study highlights the knowledge-practice gaps on frailty screening, reporting of frailty and collaboration with geriatricians. Recommendations include education on geriatric perioperative care to gain confidence in frailty assessment and consistent involvement and participation of anesthesiologists in the process of perioperative care for frail elderly. Future research should focus on the involvement of anesthesiologists in perioperative management of frail elderly on postoperative outcome; giving rise to the sub-specialization of geriatric anesthesia.

## Supplementary Information

**Additional file 1 **: **Supplement A**. The survey ‘the anesthesiologist and frail elderly’. **Supplement B**: The dataset of the survey.

## Data Availability

The authors declare that the data supporting the findings of this study are available within the article and its supplements.

## Abbreviations

AAGBI: Association of Anaesthesists of Great Britain and Ireland
ICU: Intensive Care Unit
USA: United States of America
WMO: Medical Research Involving Human Subjects Act
