# Supplementary material for: Frailty as a growing challenge for anesthesiologists – results of a Dutch national survey
Source: BMC Anesthesiol. 2021 Dec 6;21:307. doi: 10.1186/s12871-021-01528-x (PMC8647406; doi:10.1186/s12871-021-01528-x)
Supplement: Supplementary file 1 — Additional file 1 : Supplement A. The survey ‘the anesthesiologist and frail elderly’. Supplement B: The dataset of the survey. [file 12871_2021_1528_MOESM1_ESM.zip › Supplement A. Bouwhuis et al. The Survey.docx]

**Supplement A: Survey ‘the anesthesiologist and frail elderly’**

The original survey (translated into English).

| **Statements** |  |
| --- | --- |
| I feel competent to recognize a frail elderly person | True / False |
| I believe the presence of frailty should be known to the anesthesiologist | True / False |
| I believe that the presence of frailty should influence anesthetic management | True / False |
| During preassessment, I report the presence of frailty in the patient file | True / False |
| All patients >70 years of age are screened for frailty when entering the hospital | True / False |
| In my hospital, I know where to find the frailty screening in the patient file | True / False |
| In my hospital   - a geriatric team is available - the anesthesiologist is part of the geriatric team - a well implemented preoperative approach for elective frail elderly patients exists - a dedicated pathway for the elderly person with a femur fracture is used - there is adequate collaboration between anesthesiologist and geriatric specialist | For each:  yes / no / unknown |
| What is the organizational set up of geriatric consultation in your hospital?   - the anesthesiologist requests consultation of the geriatric specialist - the leading physician requests consultation of the geriatric specialist - the geriatric specialist is considered to be in the lead - unknown - other: …………………………………………… | |
| I see the increasing number of (frail) elderly patients in the nearby future as:   - a problem for society - a challenge - a gap in my knowledge and skills - a reason for emergence of an anesthesiologic sub specialization for elderly patients - not our responsibility as anesthesiologists | |
| At this moment, I work as a …   - resident, with 1 / 2 / 3 / 4 / 5 years of training - consultant, with <5 / 5-15 / >15 years of working experience - other: ……………………………….. | |
| The most of my working hours are in:   - Non-academic hospital - Academic hospital | |

This is a supplement to the article:

Frailty as a growing challenge for anesthesiologists – results of a Dutch national survey

Authors: Bouwhuis A., van den Brom C.E., Loer S.A., Bulte C.S.E.
